# Sparse Representation of Brain Aging: Extracting Covariance Patterns from Structural MRI

**DOI:** 10.1371/journal.pone.0036147

**Published:** 2012-05-08

**Authors:** Longfei Su, Lubin Wang, Fanglin Chen, Hui Shen, Baojuan Li, Dewen Hu

**Affiliations:** College of Mechatronics and Automation, National University of Defense Technology, Changsha, Hunan, People’s Republic of China; Institution of Automation, CAS, China

## Abstract

An enhanced understanding of how normal aging alters brain structure is urgently needed for the early diagnosis and treatment of age-related mental diseases. Structural magnetic resonance imaging (MRI) is a reliable technique used to detect age-related changes in the human brain. Currently, multivariate pattern analysis (MVPA) enables the exploration of subtle and distributed changes of data obtained from structural MRI images. In this study, a new MVPA approach based on sparse representation has been employed to investigate the anatomical covariance patterns of normal aging. Two groups of participants (group 1∶290 participants; group 2∶56 participants) were evaluated in this study. These two groups were scanned with two 1.5 T MRI machines. In the first group, we obtained the discriminative patterns using a t-test filter and sparse representation step. We were able to distinguish the young from old cohort with a very high accuracy using only a few voxels of the discriminative patterns (group 1∶98.4%; group 2∶96.4%). The experimental results showed that the selected voxels may be categorized into two components according to the two steps in the proposed method. The first component focuses on the precentral and postcentral gyri, and the caudate nucleus, which play an important role in sensorimotor tasks. The strongest volume reduction with age was observed in these clusters. The second component is mainly distributed over the cerebellum, thalamus, and right inferior frontal gyrus. These regions are not only critical nodes of the sensorimotor circuitry but also the cognitive circuitry although their volume shows a relative resilience against aging. Considering the voxels selection procedure, we suggest that the aging of the sensorimotor and cognitive brain regions identified in this study has a covarying relationship with each other.

## Introduction

Because cognitive decline and dementia are some of the greatest health threats to the elderly and old age itself is the biggest risk factor for developing neurodegenerative disease, studying the mechanisms and processes that contribute to aging has recently gained momentum [1]. To prevent the onset of these threats, it is necessary to identify the distribution of age-related changes in the healthy human brain [1]–[12].

It is well established that the human brain shrinks with age [3], [4], [7], [13]–[17]. Because the volume of gray matter (GM) undergoes a significant age-related decline throughout the entire brain [4], studies involving the effects of aging on GM volume occupy a unique position in this field [17], [18]. Voxel-based morphometric (VBM) studies estimate that GM loss is 0.18% per year [19]. Most white matter (WM) changes occur during advanced aging, while the volume of GM appears to be constantly reduced throughout the aging process of the human brain [8], [13], [20], [21]. Thus, the volume of GM can be used as a stable biomarker of an individual’s age [7].

Current VBM studies of age-related changes of GM in healthy subjects have reported many different and controversial findings [2], [12], [18], [22]–[24]. For example, a cross-sectional study employing a computerized volumetric analysis of MRI data found that the prefrontal GM was most sensitive to aging, although small age-related changes in volume were also observed in the fusiform, inferior temporal and superior parietal cortices [25]. A study of normal adults revealed that the bilateral insula, superior parietal gyrus, precentral and postcentral gyri, central sulcus (CS), right cerebellum, and cingulate sulcus demonstrated significant age-related loss in volume [13], while another study found the largest age-related effect in various regions of the prefrontal cortex, medial temporal lobe, and striate cortex [26]. The non-uniform nature of age-related changes, lack of samples, or deficiencies of conventional methods may lead to these different and controversial conclusions. Thus, new techniques and larger number of structural MRI images are urgently needed to determine the global spatial patterns of aging in the human brain.

Many statistical approaches have been implemented under a general linear model (GLM) framework [27]. Ranking by a two sample t-test is one of the most widely used univariate methods of the GLM framework [6]. This method can effectively select voxels that decrease in volume significantly with age, however, it does not consider the interrelationships among brain regions [28]. Unlike the univariate methods, which select discriminative voxels by focusing on only a single voxel at a time, multivariate pattern analysis (MVPA) views multiple voxels as a representation of the brain state [29]–[32]. As more of the machine learning technique has been applied to biological imaging analysis [33], [34], a number of studies have successfully employed MVPA to detect discriminative brain regions from MRI images [5], [32], [35]–[39].

In MVPA, sparse representation has recently gained popularity because of its ability to construct high dimensional data with compressed samples. In addition, many techniques based on sparse representation have been successfully applied to MRI studies. As a Bayesian extension of logistic regression, sparse logistic regression has performed well in the classification of functional MRI data [40], and the elastic net has been used to predict and interpret neural activity based on functional MRI data [41]. By iteratively solving the linear programming problem, a novel sparse representation algorithm was presented [42].

Recently, growing evidence has supported the promise of sparsity in human-brain mapping studies [43], [44]. Independent component analysis is successful in MRI studies because of sparsity and not because of independence [43]. Furthermore, several previous studies have shown that the true brain network is sparse [45]–[47], and that the brain regions that are functionally connected can be sparse as well. In addition, the age-related shrinkage of brain regions is selective, and these regions can be functionally connected, which may underlie various cognitive functions [1], [25], [48]. Based on these findings, we hypothesized that the most discriminative spatial patterns of aging may be the sparse representation of aging for the whole brain, and that this may be the physical substrate of functional decline in the human brain. However, to our best of knowledge, this is the first report to apply sparse representation-based methods to analyze structural MRI data.

Considering the advances made in sparse representation techniques and the sparse characteristics observed in age-related MRI studies, we proposed an MVPA scheme based on sparse representation to extract the spatial patterns of normal aging. This algorithm calculates sparse resolution by iteratively solving a linear programming problem. It is particularly fit for decoding tasks based on MRI data [42], [49]. One advantage of this sparse representation method is that it categorizes the selected voxels into two parts, where one part were selected for the information contained in single voxels which is directly correlated to the class information of the participants, and the other part were selected for accumulating information contained in combination of voxels, whose volume shows a covarying relationship between each other.

**Figure 1:**
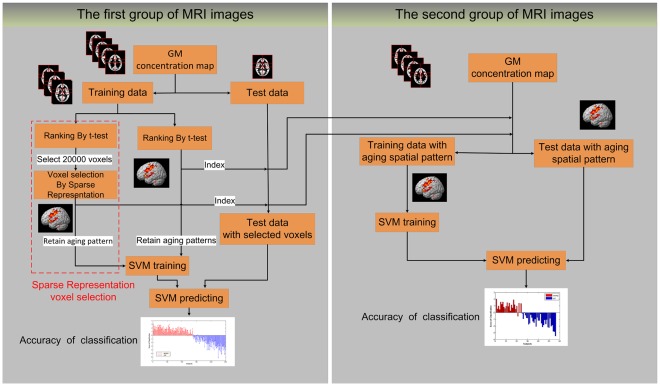
Procedure to identify discriminative voxels by the sparse representation method and t-test filter. The sparse representation method and a t-test were applied to the original GM volume map. There are 2 steps in the sparse representation method: first, filtering of the original data by a t-test, where 20,000 voxels were retained; second, the sparse representation algorithm was performed on these voxels. Next, according to the age-related classification accuracy, we fix the number of remaining voxels as discriminative patterns of aging. As a comparison, the t-test selects the same amount of voxels as aging patterns for classification. The voxel selection and SVM training were both performed using a ten-fold cross validation on the first group of MRI images. The first 1,000 voxels of the intersection of rearranged voxels in the ten folds were defined as the final spatial patterns of aging. The final spatial patterns of aging according to sparse representation and the t-test were then applied on the second group of MRI images and tested by the LOOCV.

Finally, pattern classification is used to evaluate the voxel selection results. Experimental results have demonstrated that this method may identify the covariance patterns of age-related tissue changes. The procedure of voxel selection and classification is illustrated in Figure 1.

## Results

### Classification Results Using Selected Voxels

**Figure 2:**
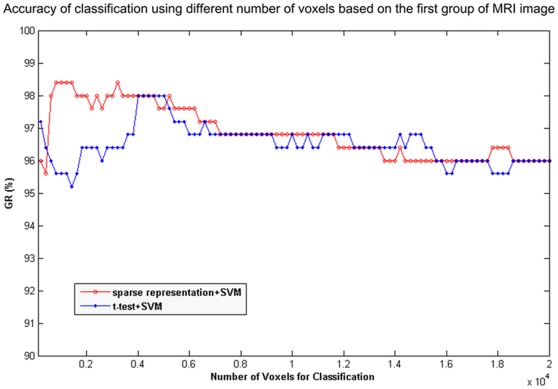
Classification results of the sparse representation and t-test filter (group 1). The voxels were ordered according to weight given by sparse representation and score of two-sample t-test. The x-axis is the number of voxels used for the classification, and the y-axis is the classification accuracy (GR).

Classification was performed using just the first few voxels selected by sparse representation. This is based on the hypothesis that the most discriminative spatial patterns of aging are sparse. Figure 2 shows the classification result using voxels selected by sparse representation according to the ordering of the voxels: a decreasing arrangement by weight as determined by sparse representation. For a better understanding of the effects of the two steps of the proposed method, the voxels arranged according to the score of a t-test filter in the first step were also used for classification. The result is displayed in Figure 2. The sparse method has two advantages over a t-test; one advantage is its high classification rate (98.4%), and the other advantage is the ability to achieve this accuracy using as few voxels as possible (about 1000 voxels).

Figure 2 indicates that generalization rate (GR) of the classification reaches its peak at 98.4% using only 1000 voxels identified by the sparse representation method, while the classification accuracy using the structural connection is 87.46% [38]. This is a very high rate of accuracy compared with the state-of-art technology. However, additional voxels can degrade the performance of the classifier. In contrast, the GR of classification based on a t-test reaches its peak when more voxels were needed. Because the proposed method includes a t-test filter, the chosen voxels are included in the voxels directly selected by a t-test when selecting for the same amount of voxels. Thus, with sufficient confidence, the second step of proposed method predominantly contributes to higher classification accuracy.

**Figure 3:**
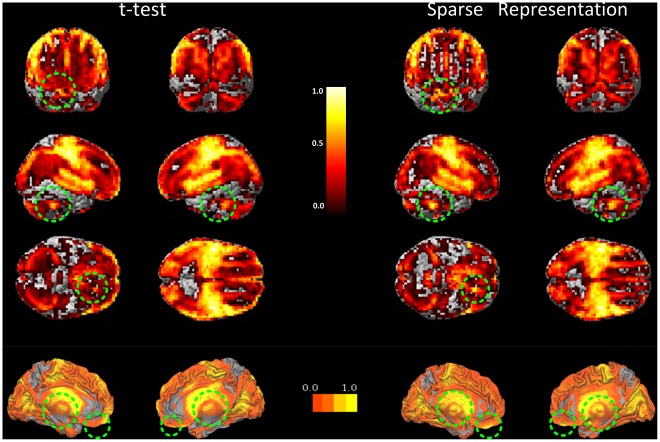
Full map of the sparse weightings and t-test values of all the 20,000 voxels used in **Figure 2**. The sparse algorithm in a recursive procedure selects 200 voxels until all of the 20,000 voxels are selected. According to the selection order, the voxels were given weightings from 1.00, 0.99, 0.98, …, 0.01. The t-test score was generated by the same procedure. The voxel selection was implemented using the ten-fold cross-validation strategy, where ten groups of voxel weightings were generated. The mean of the ten groups of weightings was defined as the final weighting.

We aimed to providing an overview of the weightings of the entire brain, and thus, projected the t-test values of the first 20000 voxels and weightings of the sparse method onto the human brain map. These results are shown in Figure 3. In particular, we focused on the weightings of the brain regions in green circles. These regions were weighted more by the sparse method and will be further discussed in our study.

When used as a classifier, SVM will give each subject a score according to its distance from the separating hyperplane. The SVM scores were closely related to chronological age. The Pearson correlation coefficient of the SVM score and chronological age has been studied and found to be r = 0.9339 for sparse representation + SVM and r = 0.9279 for t-test + SVM.

**Figure 4:**
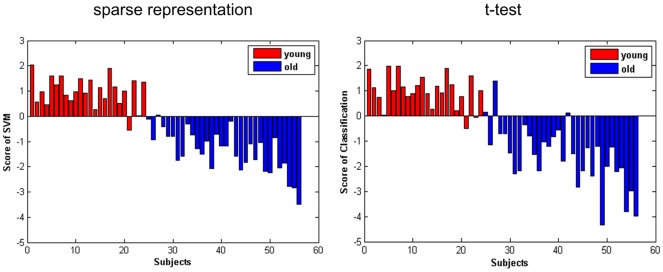
Score of the SVM obtained in group 2 based on the spatial patterns of aging identified in group 1. The results displayed in the left figure were obtained on the second group of MRI data by SVM. Voxels used for classification were the final spatial patterns of aging which were generated on the first group of MRI data; the right results are SVM score using voxels obtained by two-sample t-test.

The final covariance patterns constructed by the first group of MRI data were then applied to the second group of MRI images. These classification results are shown in Figure 4. The graph on the left is classification results of the spatial pattern selected by the sparse representation (GR: 96.4%, SS: 95.8%, SC: 96.8%), while the graph on the right represents the classification results according to a t-test (GR: 91.1%, SS: 91.7%, SC: 90.6%).

### Discriminative Spatial Patterns of Aging

**Figure 5:**
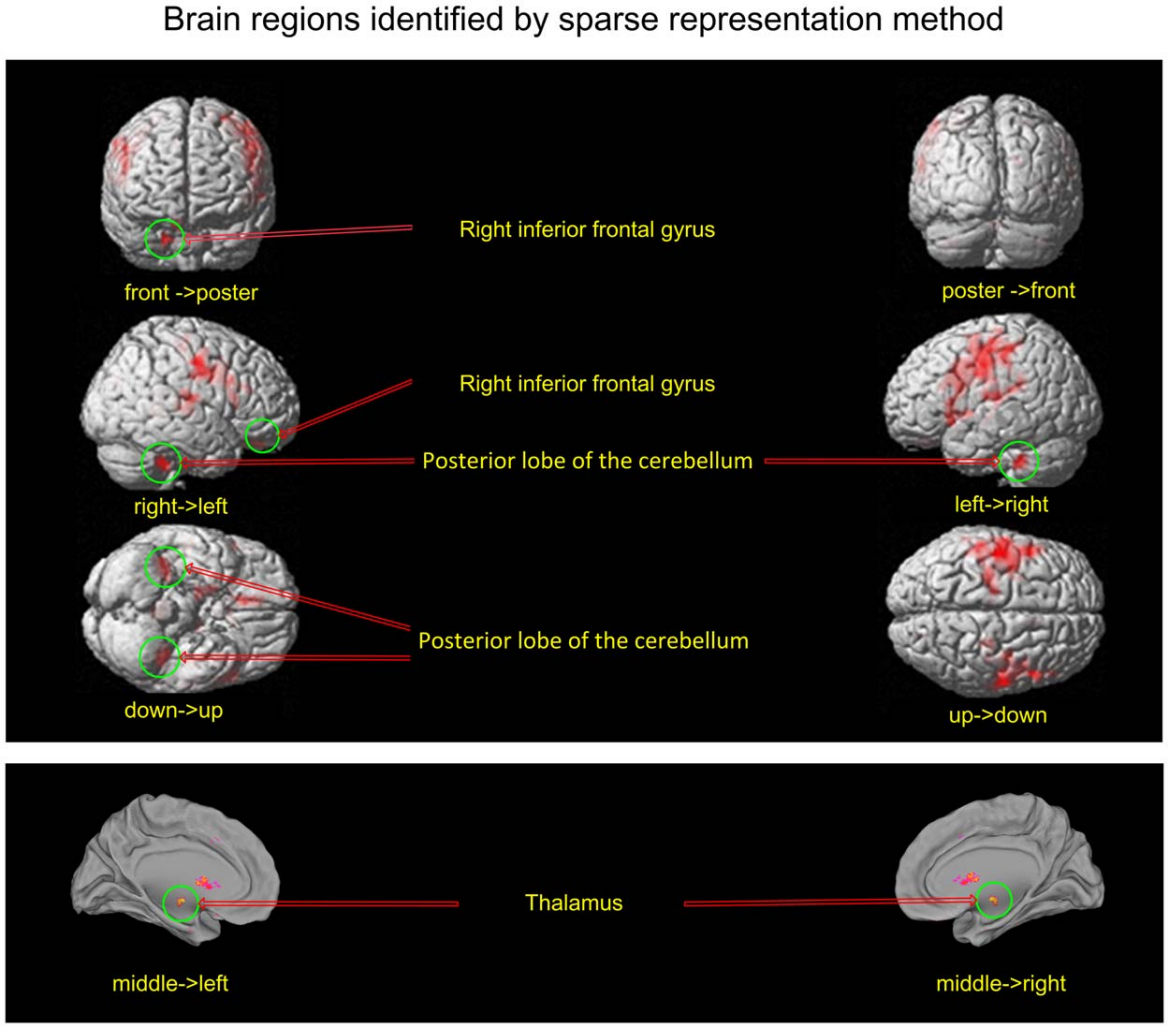
Locations of the representative brain regions of informative patterns identified by the sparse representation method. The first 1000 voxels of spatial patterns identified by sparse representation were projected on the original human brain map. The sparse representation method selected human brain regions that included regions selected by a t-test filter, and were more widespread. The regions in the green circles were only identified by sparse representation.

**Figure 6:**
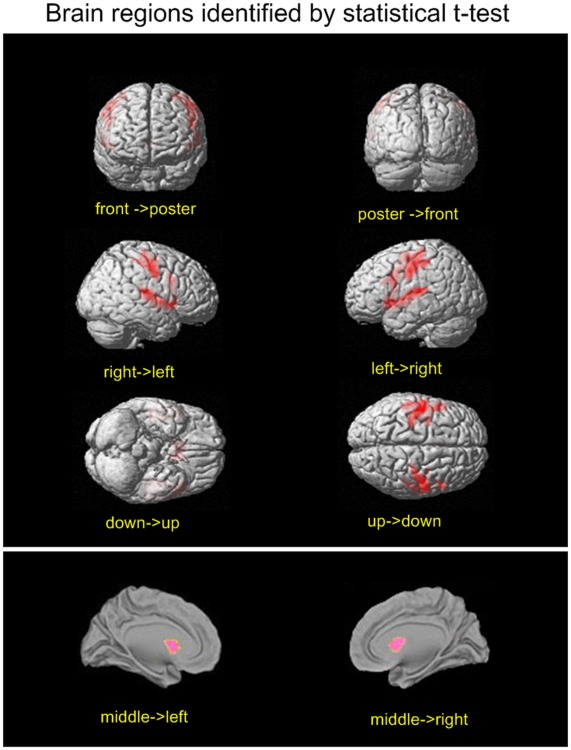
Locations of the representative brain regions of informative patterns identified by t-test filter. The first 1000 voxels of brain regions identified by a t-test filter were projected on original brain map.

Figure 5 shows the final spatial patterns of aging, which were extracted by sparse representation with the goal of facilitating analysis. The representative regions were defined from the spatial patterns according to the cluster size. Their anatomical labels and Montreal Neurological Institute (MNI) coordinates obtained by the xJview MATLAB toolbox are summarized in Table 1. For a comparison with the final covariance patterns, Figure 6 shows the results of the statistical t-test between GM volume of the young and the old.

**Table 1:** The locations of the voxels that were selected by the sparse representation and statistical t-test.

Cluster	Voxels	Hemisphere	Brodmann Area	MNI Coordinate		
				X	Y	Z
Clusters Selected by Sparse Representation						
Precentral & Postcentral gyri	404	L	2/3/4/6/13/40/41	−44	−4	18
	316	R	3/4/6/13/41	48	−8	16
Middle frontal gyrus	20	L	6	−30	−4	58
Caudate	83	L&R		0	11	5
Posterior lobe of the cerebellum	12	L		−36	−40	−40
	11	R		40	−40	−40
Inferior frontal gyrus	12	R	11/25	12	34	−24
Thalamus	19	L&R		−12&14	−4	5
Clusters Selected by Statistical t-test						
Precentral & Postcentral gyri	449	L	2/3/4/6/13/40/41	−46	−9	21
	344	R	3/4/6/13/22/41	50	−8	24
Middle frontal gyrus	31	L	6	−29	−4	60
Caudate	137	L&R		0	12	4

By comparing Figure 5 with Figure 6, we can distinguish between the brain regions selected by the second step of sparse representation from those selected by a t-test filter. In addition to the four clusters selected by a t-test, the other four clusters were selected by sparse representation in the second step. These clusters contain accumulating information regarding the covarying relationship of the voxels. Only these clusters exhibited increases in classification accuracy, which can be seen in Figure 2 and Figure 4.

The locations of the clusters identified by the proposed method and a t-test are displayed in Table 1. The voxels selected considering the relationship between the volume in a single voxel and age are concentrated in clusters confined to regions near the CS, which include the precentral and postcentral gyri, middle frontal gyrus and caudate nucleus. In contrast, the voxels selected by the second step distributed in more regions across the entire brain. In addition to the aforementioned brain regions, subcortical brain regions including the posterior lobe of the cerebellum, thalamus, and right inferior frontal gyrus were selected. These regions led to the difference in classification accuracy between a t-test and sparse representation as shown in Figure 2. This issue will be addressed in further detail in the discussion section.

**Figure 7:**
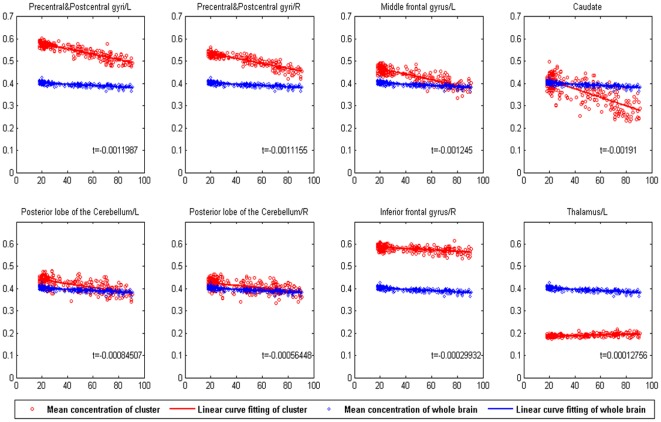
Relationship between the mean volume of clusters and subject age (group 1). The red circles represent the mean volume of all the voxels in one cluster for all of the subjects in the first group; the blue diamonds represent the mean volume of the entire brain (GM) for all of the subjects in the first group. The red and blue lines are the results of the linear curve fitting corresponding to the red circles and blue diamonds. The “b” represents the coefficient of linear regression of the mean cluster volume. The hypothesis t-test of the coefficients was implemented at a significance level of 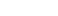.

Subsequently, eight clusters were then selected from the entire brain. The mean volume of each cluster of all the 290 subjects in the first group of MRI images and the ages of the subjects are plotted in Figure 7. A linear regression model was then used to fit the mean volume and chronological age of all the subjects. In addition, a hypothesis test on the regression coefficients in the linear regression was performed. The coefficients are shown in Figure 7, with significance of 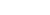. From Figure 7 we observed that the volume of the precentral and postcentral gyri, middle frontal gyrus, and caudate nucleus are significantly reduced with a coefficient of 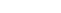. This indicates that these regions were selected because of their direct relationship with the age. The posterior lobe of the cerebellum, thalamus, and inferior frontal gyrus did not reveal a significant volume decline, with a coefficient of 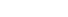. It is not necessary to confirm that these voxels were not affected by aging. These regions were selected based on their covarying relationship of voxels, which corresponded with the second step of the sparse representation method. Thus, the sparse representation method can successfully select for subtle and covariance changes in the human brain.

**Figure 8:**
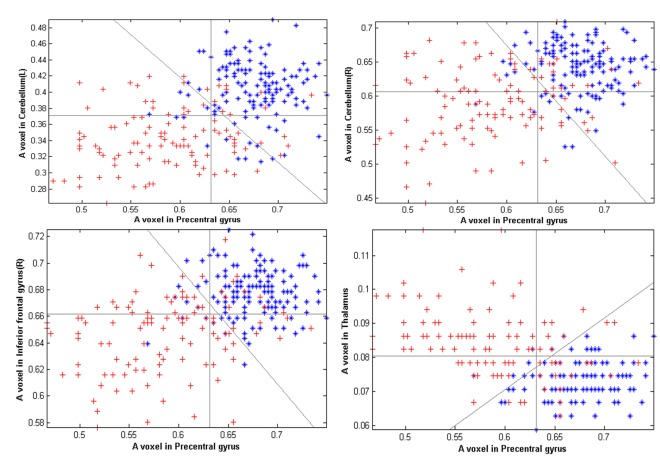
Contribution of the correlation between a voxel in the precentral gyrus and voxels in the cerebellum, inferior frontal gyrus and thalamus. The x-axis represents the gray value of a representative voxel in the precentral gyrus that corresponds with the 250 subjects used for classification, and the y-axis represents the gray value of a representative voxel in the left cerebellum (top-left), right cerebellum (top-right), right inferior frontal gyrus (down-left) and thalamus (down-right). The blue stars and red crosses represent the young cohort and old cohort samples in the first group of MRI images, respectively. The three gray lines in each subplot are discriminant boundaries estimated by the SVM which correspond with the x-axis, y-axis and combination of both. It is noteworthy that the voxel in the precentral gyrus is more discriminative (84.4%) than the voxels in the clusters described above (the left cerebellum 82.0%, right cerebellum 72.0%, inferior frontal gyrus 77.6%, and thalamus 80.4%), but the discrimination ability derived from combination of a voxel from the precentral gyrus and a voxel form the specific cluster is even higher (the left cerebellum 87.6%, right cerebellum 86.8%, inferior frontal gyrus 89.2%, and thalamus 89.2%).

For a better understanding of the importance of the correlation of voxels selected by the sparse method, refer to Figure 8. One voxel from each cluster described above was selected to investigate its discriminative ability just as Yamashita did in [40].

## Discussion

In this study, sparse representation-based voxel selection and classification were combined to extract the covariance patterns related to normal aging. Based on a thorough understanding of the sparse representation method, we analyzed our voxel selection scheme and utilized the advantages of the sparse representation method and overcame its disadvantages. According to the results of this study, we re-evaluate the spatial patterns of normal aging. In addition to the brain regions generally accepted as important nodes of the sensorimotor system, other brain regions that could be implicated in cognitive decline were also identified by the second step of the proposed method, although these brain regions did not show apparent volume reductions with aging. To our knowledge, this is the first report that reveals the coupling between the aging of sensorimotor and cognitive function using T1-weighted structural images with MVPA.

### Clusters that Covary with Age

The precentral and postcentral gyri, and the caudate were identified by the first step of sparse representation for their volume decline in single voxels. Most of the selected voxels were located in these regions, in particular, within the areas near the CS, which have also been referred to as aging sensitive brain regions in previous studies [3], [8], [10].

For the t-test, most of the selected voxels (approximately 80%) are concentrated in these areas which result in the most significant volume reduction. In contrast, comparative fewer voxels (approximately 70%) distributed in these areas for the proposed method, which had selected a greater number of voxels in the precentral gyrus, which indicates that the method weighted the precentral gyrus more heavily than the postcentral gyrus. In healthy adults, the thickness of the precentral sulcus is greater than that of the postcentral sulcus, and the cortical thinning of the precentral sulcus with age is more significant than that of the postcentral sulcus [14], [15], [50]. These localizations are well accepted as sensorimotor regions. In addition, our study provided further support by confirming that these regions are very sensitive to aging.

Fourteen percent of selected voxels for the t-test and eight percent for the proposed method were focused in the caudate nucleus. This confirmed another important brain region that has a significant volume decline with age. The caudate nucleus has been previously linked to an age-related decline and changes in motor performance [18], [51], which indicates that this region may be one of the stable patterns of aging.

Sensorimotor-related brain regions, including the precentral, postcentral gyri, and the caudate, have been shown to be affected by aging in some studies focused on sensorimotor performance and age-related brain alterations [9], [52], [53]. The left precentral and postcentral gyri, right precentral and postcentral gyri, left middle frontal gyrus, and caudate are critical for sensorimotor tasks. We evaluated the developing trend of these clusters with the individual’s age. Our results are shown in Figure 7. The results of the hypothesis tests in the linear regression for these clusters can be observed. In addition, the coefficients of the linear regression for these clusters are shown in Figure 7 (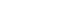) with a significance of 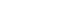. Thus, these clusters show a significant volume reduction with age.

### Clusters that Covary with Other Clusters

By comparing the spatial distributions selected by the proposed method and t-test (Figure 5 and Figure 6), we conclude that the cerebellum, thalamus and prefrontal cortex were detected by the second step of the proposed method for the consideration of information contained in covarying relationship of voxels in different locations. The GM of the posterior lobe of the cerebellum, inferior frontal gyrus, and thalamus did not display a significant volume decline, as shown in Figure 7. The changes in these clusters were so subtle that they were undetectable using univariate methods. However, the new algorithm was able to detect these changes when fewer voxels were considered. This confirms the efficiency of the new sparse representation method.

The cerebellum is commonly affected by aging, although its volume reduction is not as extensive as the cerebrum [16], [25], [54]. Inside the cerebellum, atrophy occurs more rapidly in some regions compared with others. Consistent with the results of our study, shrinkage has been shown to occur predominantly in the posterior lobe [13], [16], [55]–[58]. The posterior lobe of cerebellum is generally involved in motor control and coordination. Our results support the idea that a loss of volume in the posterior lobe is related to the lack of mobility observed in the elderly. Moreover, a volume decline in the precentral and postcentral gyri, which cover most sensorimotor brain regions, may be closely associated with changes in the posterior lobe of the cerebellum [59]. The interaction between these brain regions may be a physical substrate that underlies the sensorimotor slowing that occurs with aging.

Currently, the role of the thalamus in the aging process remains controversial. Several studies have found that GM volume in the thalamus declines with age [11], [28], while other studies have suggested that the volume actually increases with age [5]. We have computed the average volume of voxels that correspond to the selected spatial patterns and compared all the spatial patterns young and old subjects. The volume of 90.3% voxels in the identified spatial patterns decreased in the old cohorts comparing with the young, while only 9.7% voxels increased, and the decrease was predominately found in the subcortical structures, particularly, the left thalamus.

There is no age-related study that suggests that the right inferior frontal region might be a biomarker of aging in the healthy human brain, although this region has been considered essential in response inhibition [60]. Moreover a growing amount of evidence indicates that this stopping behavior is markedly slowed during adulthood [61]. This implies that the right inferior frontal region contains significant aging information. Furthermore, this region was also identified by the proposed method but not a t-test. Voxels in this region were fewer, and the volume decline was not apparent and undetectable using the t-test. Thus, this region may be selected for its correlating covarying relationship with other brain regions.

The cerebellum, the thalamus and the right inferior frontal gyrus have been reported as three important nodes of the cognitive network in the human brain [62]–[64]. Regions of prefrontal cortex and the connections between the sensorimotor cortex, thalamus and cerebellum are believed to construct the cognitive circuitry [62]. The precentral and postcentral gyri, right inferior frontal gyrus, thalamus and cerebellum were all detected by the proposed method. This result suggests that aging affects cognitive circuitry in the human brain. A correlation between cognitive decline and aging has already been established [65]–[67]; however, additional studies are required to confirm the brain network responsible for these phenomena. In this study, we detected specific brain regions that might be involved in this network. Our results provide supplementary evidence for the cognitive decline.

**Figure 9:**
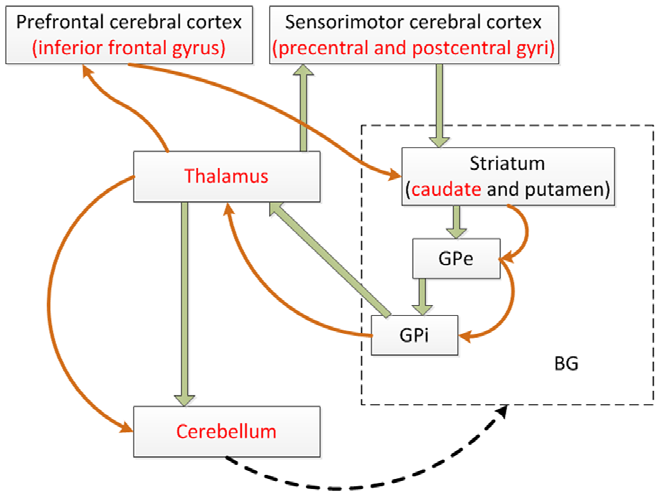
Brain regions extracted by the new method distributed over the sensorimotor and cognitive circuitry in the human brain. The red font brain regions are identified in our study. The green arrows represent the sensorimotor functional circuitry, while the brown arrows represent the cognitive circuitry. The black dash arrow represents the newly found in [59]. BG: basal ganglia, GPi: internal globus pallidus, GPe: external globus pallidus.

Couplings between sensorimotor and cognitive circuitry in the human brain have been recognized in many studies [64], [68]–[71]. The aging of these two circuits has also been suggested to be interactive [66], [72]–[74]. The basal ganglia (BG) which consists of the caudate and putamen, globus pallidus, and substantial nigra, is a critical cohesive functional unit in the human brain. The BG is not only highly related to sensorimotor tasks of human brain, but also involved in cognitive function [59], [64], [75]–[80]. The cerebellum and thalamus are always included in BG-mediated circuitry [59], [62], [75]–[77], [79]. The critical nodes of the sensorimotor and cognitive circuitries are shown in Figure 9 [59], [64], [77]–[79]. The caudate, cerebellum and thalamus are shared by both the sensorimotor and cognitive circuitry, whose changes may be the substrates of the covarying relationship between sensorimotor and cognitive decline. Our study provids further evidence for the coupling between sensorimotor and cognitive function from the aspect of structural neuroimaging. Univariate methods, such as the t-test under GLM framework, can effectively identify the brain regions whose volume reduced rapidly with aging. Conversely, in the MVPA methods, sparse representation can extract brain regions whose volume displays resilience against aging by evaluating the accumulating information contained in the covarying relationship of voxels. Our study complements previous based aging researches based on functional connectivity and facilitates the analysis based on functional network and circuitry [6], [81].

### Consideration of the Voxel Selection Method

Our voxel selection consists of two steps: (1) a t-test filter under the GLM framework that is fit to localize discriminative brain regions and, (2) sparse representation algorithm that selects a combination of distributed voxels for accumulation information [42]. The first step of our approach involves the ability to bundle discriminative voxels into clusters for the volume decline of single voxels, and the second step selects a combination of clusters containing information of covarying relationship among the voxels. The resulting improvement in classification accuracy may be achieved by the second step because of the volume decline of brain regions and the covarying relationship between the aging clusters across the entire brain.

The sparse representation algorithm can effectively solve the over-fitting problem, but it presents the additional challenge of over-pruning, which occurs when the representation algorithm selects only several representative voxels from each discriminative cluster. The representative voxels may be different when the algorithm reruns on the same data. Because the basic brain function units are voxel clusters, the voxels selected by sparse representation cannot effectively reflect the stable distribution of brain aging. Therefore, it is difficult to construct stable, functionally related spatial patterns of aging in the human brain by directly using the voxels selected by the sparse representation algorithm. To address this issue, a ten-fold cross validation was employed, and the intersection of all ten groups of rearranged voxels was reconsidered. In this intersection, the first 1000 voxels, which were selected from a spread range, were defined as the final spatial patterns of normal aging. Thus, It could be a more stable biomarker of aging and correct the over-pruning problem.

The final spatial patterns of aging that were derived from the intersection of the ten groups of voxels selected in the ten-fold cross validation contain the aging information of all the MRI images in the first group. To avoid using test data for classifier training and test the generalizability of the spatial patterns [24], the final spatial patterns of normal aging were applied to the second group of MRI data. Using only the spatial pattern, 96.4% of the subjects were correctly classified. The MRI images in the second group were scanned with a different MRI machine which indicates that our new method is not sensitive to equipment changes.

Using only 1,000 of the total 35,499 voxels, we can successfully distinguish the young from the old. Thus, we confirmed the hypothesis that the spatial patterns of aging in our study could be sparse representation of aging for the entire brain. Moreover, the correlation between the sensorimotor and cognitive networks is also highlighted by this study.

The sparse representation method is a supervised method, which can take either the discrete or continuous variable as the label. One limitation of this study was that only the binary class label (1 and −1) was adopted in the sparse representation-based voxel selection. This limited the ability of the sparse method to explore additional aging information. Next, we will attempt to take the chronological age as the label of the sparse representation-based method. Although this voxel selection method provides good performance on data sets with different parameters for scanning, it is important to evaluate this method with large size of samples. Additionally, the brain function features derived from functional MRI will be acquired to further validate the results of this current study.

## Materials and Methods

### Ethics Statement

Approvals for public sharing were obtained from all the subjects.

### Participants

Two groups of MRI images were selected from two databases. One group included MRI images of 290 subjects which were downloaded from the open access series of imaging studies (OASIS) website (http://www.oasis-brains.org). The initial data set in OASIS consists of a cross-sectional collection of 416 subjects aged 18 to 96 years [82]. One hundred suffered with AD and 26 not successfully segmented were excluded from our study. At last, 290 subjects were retained. 138 young healthy subjects between the ages of 18 and 30 (22.5±3.0) and 112 old healthy individuals between the ages of 50 and 91 (69.1±11.7) were selected for pattern classification. The young subjects were recruited from the community of Washington University while the old subjects were recruited from a longitudinal pool at Washington University Alzheimer Disease Research Center (ADRC).

For the second group, 56 healthy subjects including 24 young subjects (22.7±3.3 years old, 19–30 years) and 32 old subjects (62.2±8.2 years old, 50–79 years) were selected from the website of 1000 Functional Connectomes project (http://fcon_1000.projects.nitrc.org/fcpClassic/FcpTable.html). There are 86 initial data sets in this database. One not successfully segmented and twenty nine aged between 30 and 60 years were excluded from this study. Only 56 subjects were retained. All of the subjects were recruited from International Consortium of Brain Mapping (ICBM) dataset. All of the subjects had no history of neurological or psychiatric disorders.

Details regarding both participant groups are shown in Table 2.

**Table 2:** Demographic characteristics of the subjects.

	Young adults				Older adults			
	Mean (years)	SD (years)	Range (years)	Number of participants	Mean (years)	SD (years)	Range (years)	Number of participants
Group 1	22.5	3.0	18–30	138	69.1	11.7	50–91	112
Group 2	22.7	3.3	19–30	24	62.2	8.2	50–79	32

### Imaging Protocol

In group 1, T1-weighted structural magnetization prepared rapid gradient echo (MP-RAGE) images were obtained with the following parameters: TR = 9.7 ms, TE = 4 ms, slice thickness = 1.25 mm, slice number = 128, flip angle = 10°, and in-plane resolution = 256×256 (1 mm×1 mm). For each subject, 3–4 T1-weighted structural images were obtained on a 1.5 T Vision scanner (Siemens, Erlangen, Germany) during a single image session. In this study, one T1-weighted MRI image was randomly selected for each subject.

In group 2, the T1-weighted structural MRI images were acquired with the following parameters: TR = 22 ms, TE = 9.2 ms, slice thickness = 1 mm, flip angle = 30° and in-plane resolution = 256×256. The images of the 56 subjects were obtained from the Functional Connectomes project and the T1-weighted structural MRI images were used for this study. All images were collected using a Siemens Sonata 1.5 T MRI scanner.

### Data Preprocessing

Data preprocessing was performed using SPM8 (http://www.fil.ion.ucl.ac.uk/spm/). First, the new segment procedure was used to segment the MRI images into six partitions including GM, WM, cerebrospinal fluid (CSF) and three other background partitions based on a modified mixed model cluster analysis technique. The new segment procedure is generally more robust than using the “segment” button. Next, a template was generated from the entire image dataset using the diffeomorphic anatomical registration by exponentiated Lie algebra (DARTEL) technique [83] which matches the GM and WM to each other. Finally, GM images were spatially normalized to the template that was created in the second step and then smoothed by an isotropic Gaussian filter with an 8 mm full-width half-maximum kernel.

### Voxel Selection

The sparse representation method was introduced for voxel selection [42]. The proposed method includes two steps: the t-test filter and a sparse representation algorithm. To achieve the cluster effect and fix the computational problem faced by the second step, the number of selected voxels set for classification cannot be set too large. Therefore, we filtered the original data using a t-test and retained 20,000 voxels in the first step. In the second step, the sparse representation was computed on the retained voxels. The purpose of the first step was to select voxels by considering the relationship between the volume in a single voxel and age, while the second step aimed at selecting bundles of voxels based on accumulating information contained in the covarying relationship of voxels in different locations [42].

The algorithm of sparse representation used in this study is presented. The model of the sparse representation algorithm can be described with the following equation:
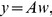
(1)where, 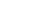
is a given label vector for training. When the algorithm is used for two-class pattern classification, every element of y is either 1 or −1. 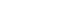 is a basis matrix in which each column represents the corresponding voxels of different subjects, and each row represents all of the voxels in the same subject. 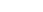 is an unknown weight vector. The object of sparse representation algorithm is to find the weight vector 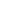 that satisfies equation (1) and simultaneously is as sparse as possible.

Consider the following optimization problem:
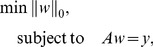
(2)The number of nonzeros of a vector is defined as 0-norm. 0-norm of 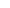 is the sparsest solution of equation (1). However, it is difficult to get the solution of problem (2). Thus, we consider an alternative optimization problem:
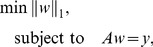
(3)The sum of the absolute value of a vector is defined as 1-norm. The optimization problem in (3) is a linear programming problem that can be easily solved. Although the solution to equation (3) is often not the sparsest and is not the same as the solution to equation (2), the two solutions can be viewed as equivalent under some conditions. Because it is too difficult to achieve sufficient conditions under which the two sparse solutions are equal, a new method based on probability was adopted. If the two solutions can be viewed as equal with a high probability (e.g., 0.95), then we can use the solution to equation (3) instead of the solution to equation (2). Another advantage of using solution to equation (3) instead of that to equation (2) is that the 1-norm solution is insensitive to noise. Therefore, 1-norm is a good measure of sparsity.

To solve the optimization problem presented by equation (3), we converted it into another format (see below) and defined new non-negative variables 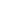 and 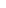, where 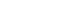 and 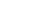,
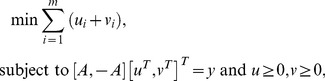
(4)Now, the problem has been converted into a typical non-negative linear programming problem, and we can easily determine 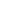 then 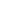. The value of the weight vector 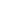 represents the weight of the corresponding column of 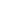. When the number of samples is not sufficiently large, a single optimization procedure cannot reflect the importance of each voxel, and several additional steps are needed which are described in the Text S1.

### Classification and Cross-Validation

The SVM was used to implement the age-related classification. SVM belongs to a learning system that is based on advances in statistical learning theory, and it seeks the separating hyperplane with the maximal margin and minimizes the structural risk. This technique works particularly well when the number of training samples is small but the feature number is large [84]. In this study, the toolbox called Spider for MATLAB, which implemented the SVM, was used for classification. This toolbox is available for free on the website for academic purpose (http://www.kyb.mpg.de/bs/people/spider/). All of the programs used in our study were implemented in MATLAB 7.8.0 (R2009a, The Mathworks, Natick, Massachusetts, United States). Details regarding the SVM used in our study are shown as:
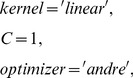
(5)The classification accuracy can be measured by generalization rate (GR), sensitivity (SS) and specificity (SC). Here, SS is defined as the proportion of correctly predicted young subjects, while SC represents the proportion of correctly predicted old subjects. The proportion of all subjects that were correctly predicted is evaluated by the GR. The Formula are shown below:
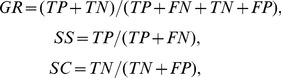
(6)where TP is the number of the young subjects correctly predicted; FN is the number of the young classified as the old; TN is the number of the old subjects correctly predicted; FP is the number of the old classified as the young.

To solve the problem in the situation that data are scarce, M-fold cross-validation uses part of the available data to learn the model, and the rest to test it. We split the data into M roughly equal-sized parts. For the *m*th part, we select voxels and learn the model to the other M-1 parts of the data, and calculate the prediction error of the learnt model when testing the *m*th part of the data. We do this for m = 1, 2, …, M and rearrange the voxels once for each m. Typical choice of M is 10. The case M = N is known as leave-one-out cross-validation (LOOCV), where N is the number of data points. In this study, M were 10 for group 1, while N for group 2. Because there were only 56 subjects in the second group, LOOCV was employed to confirm the accuracy of the classifier.

For the first group of MRI images, ten-fold cross-validation was implemented to confirm the results of the voxel selection methods and the accuracy of the classifier. We have obtained one group of rearranged voxels for each fold, and then ten groups of rearranged voxels were generated.

The first thousands of voxels in the intersection among the ten groups of rearranged voxels were chosen as the final aging spatial patterns. To confirm the robustness of the sparse representation method, we applied the spatial patterns of aging that were selected from the first group of MRI images to the second group of MRI images for classification.

## Supporting Information

Text S1 **The details about the sparse representation algorithm.**
(DOC)
