# Supplementary material for: Sparse Representation of Brain Aging: Extracting Covariance Patterns from Structural MRI
Source: PLoS One. 2012 May 8;7(5):e36147. doi: 10.1371/journal.pone.0036147 (PMC3348167; doi:10.1371/journal.pone.0036147)
Supplement: Text S1 — The details about the sparse representation algorithm. (DOC) [file pone.0036147.s001.doc]

**Sparse Representation Algorithm**

Sparse representation based voxel selection algorithm:

1. For $t=1,2,...,T$, (in this study, we choose $T$= 200 empirically) perform steps 2 to 4.
2. Using matrix $A^{t},(A^{1}=A)$, and the label $y$, perform steps 2.1-2.2 for $k=1,2,...,R$ times (in this study, we choose $R$= 300 empirically).

2.1: Randomly choose $q=0.1n$ ($n$=rows of $A$) rows from $A^{t}$ to a construct submatrix $A_{k}$, corresponding $q$ entries of $y$ forms $y_{k}$.

2.2: Solve optimization problem

$\min\left\|w\right\|_{1}\text{, subject to }A_{k}w=y_{k}$ S.1

We denote the solution of equation S.1 as .

1. Let

$w=\left|\frac{1}{300}\sum_{k=1}^{300} w^{k}\right|$ S.2

1. According the weight vector $w$, the 100 voxels with highest elements are selected. Column index of these 100 voxels are defined as . After these 100 columns removed from $A^{t}$, the remaining columns form $A^{t+1}$.
2. is the new rearranged index of the 20000 voxels.
